# Understanding and using the meaning of statements in a bio-ontology: recasting the Gene Ontology in OWL

**DOI:** 10.1186/1471-2105-8-57

**Published:** 2007-02-20

**Authors:** Mikel Egaña Aranguren, Sean Bechhofer, Phillip Lord, Ulrike Sattler, Robert Stevens

**Affiliations:** 1 School of Computer Science, University of Manchester, Manchester, UK; 2 School of Computing Science, University of Newcastle, Newcastle, UK

## Abstract

The bio-ontology community falls into two camps: first we have biology domain experts, who actually hold the knowledge we wish to capture in ontologies; second, we have ontology specialists, who hold knowledge about techniques and best practice on ontology development. In the bio-ontology domain, these two camps have often come into conflict, especially where pragmatism comes into conflict with perceived best practice. One of these areas is the insistence of computer scientists on a well-defined semantic basis for the Knowledge Representation language being used. In this article, we will first describe why this community is so insistent. Second, we will illustrate this by examining the semantics of the Web   Ontology Language and the semantics placed on the Directed Acyclic Graph   as used by the Gene Ontology. Finally we will reconcile the two representations, including the broader Open Biomedical Ontologies format. The ability to exchange between the two representations means that we can capitalise on the features of both languages. Such utility can only arise by the understanding of the semantics of the languages being used. By this illustration of the usefulness of a clear, well-defined language semantics, we wish to promote a wider understanding of the computer science perspective amongst potential users within the biological community.

## 1 Background

In this paper, we explain the role of a Knowledge Representation (KR) language's semantics. To illustrate the utility of language semantics we will use it to explore the reconciliation of the representations used for the Gene Ontology (GO) [1] and that used for the ontologies represented in the W3C recommendation Web Ontology Language (OWL [2]). A language's semantics is often a great concern to computer scientists, a concern that is sometimes lost on biologists. The goal of this paper is, therefore, to explain the role of language semantics to a community outside computer science (this albeit anecdotal evidence is built up over many years of teaching and tutorials in this domain between the two disciplines). In the text of this document **Boldface** font is used to refer to objects and logical keywords within an ontology and ***Italics Boldface ***font for terms that have a definition available in the glossary (see Additional file 1).

Different ***knowledge representation languages ***provide different means to make statements about the knowledge to be captured in different ways. The semantics of these languages tell both humans and computers how to interpret statements made in those languages. Different languages have varying expressivity and computational properties, hence the corresponding tools can offer different querying and ***reasoning ***mechanisms; consequently there is often a need to exchange between languages to take advantage of their characteristics. For example, the Web Ontology Language OWL-DL [3] comes with rather high expressivity and some powerful reasoning services. As a consequence, we can annotate data using terms (and expressions built from these terms) whose meaning is defined in some OWL-DL ***knowledge base***, usually called an "ontology", and then use a software application called a reasoner to query that data. The reasoner will take into account the definitions of the terms when answering queries, thereby providing flexible access to that data. When translating a knowledge base from one language to another, we have to make sure that the knowledge captured in statements in one language is changed as little as possible when transforming them into statements in another language. Hence, the semantics of one language needs to be reconciled with the semantics of the other.

The GO has become the *de facto *standard for describing the principal attributes (the molecular function, biological process, and cellular component) of knowledge about gene products across many databases [1,4]. It succeeds in the major aim of an ontology in providing a common, shared understanding of the concepts used to describe those attributes–for humans. It does this by providing terms used to label those concepts as well as natural language definitions of those terms.

GO is part of an umbrella project that encompasses many other bio-ontologies called Open Biomedical Ontologies (OBO [5]). GO uses a knowledge representation language developed in-house–based on the Directed Acyclic Graph (DAG) [4]. The DAG is a common-place representation across computer science and other disciplines. What the edges and nodes in the DAG mean, their semantics, is determined by the specific user community. In some graphs, for example, a node represents a railway station, an atom, *etc*. As we will see in Section 4 there is a particular meaning to the edges and nodes used in representing GO, which have been determined by the GO Consortium. The GO's DAG is encoded using a syntax also developed by this group. The DAG has the tremendous advantage of simplicity and this has been a factor in enabling the Gene Ontology to develop to its current pre-dominant status [6].

GO's DAG is available in different formats, including MySQL tables, XML and OWL [7]. The most commonly used format is, however, the OBO file format, which is shared by most of the other OBO bio-ontologies [8].

The OBO file format not only enforces the syntax the OBO files should have, it also provides a set of elements that can be used to define semantics such as **domain**, **range**, **is_symmetric**, **is_cyclic**, **is_transitive**, *etc*. GO's DAG can be represented in the OBO file format, making use of a subset from all the possible elements available. Other bio-ontologies make use of other elements, and all those bio-ontologies (GO and other OBO bio-ontologies) are compliant with the OBO file format.

The OBO site states that submitted ontologies can be presented in the OBO file format (including GO's DAG) or in OWL. Being a collection of bio-ontologies, it would be useful to be able to translate ontologies between the two formats. Indeed, this has already been attempted in both the current version of DAG-Edit [9] and its successor, OBO-Edit [10], the COBrA ontology editor [11] and as an initial step in the Gene Ontology Next Generation (GONG) project [12,13].

The primary purpose in this paper is not to present a translation of the DAG and OBO formats into OWL, but to show how such a translation is achieved. Such translation has already been done by the Gene Ontology consortium themselves [14]. We use the case study here as an illustration of the use of a language's semantics to achieve the translation and in doing so show how a strict semantics is very important. In doing this, in Section 2 we explain why computer scientists, in particular, like to have a well-defined semantics in their knowledge representation languages. In Sections 3 and 4 we outline the semantics of GO's DAG representation and that of OWL. In Section 5, we attempt to reconcile the two representations. Section 7 describes the implementation of this translation.

## 2 Why do computer scientists care so much about semantics?

**Figure 1:**
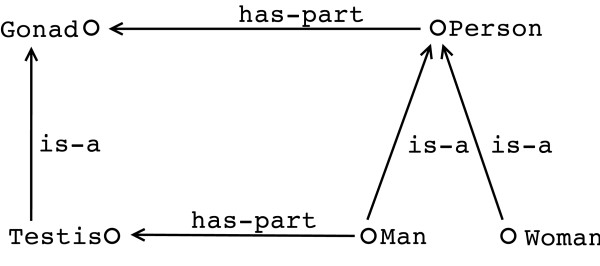
An example toy ontology of Person. The ontology takes a very simplified view of biological reproduction, for the sake of clarity.

The knowledge representation community within computer science has the aim of representing knowledge in a form both understandable by humans and one that is computationally amenable. Computers, of course, do not have the same facility to "understand" knowledge captured in an ontology as do the human users of that ontology. To a computer, the term labeling a concept is not comprehensible. For illustration (see Figure 1) we will use a deliberately simplistic ontology that is by no means biological. For this article a toy example is able to convey the point of semantics more easily than a "true" biological example that would obscure the message. Taking the example in Figure 1, a human might read the information in this representation as saying that "an instance of **Person** is either a **Man** or a **Woman**, but not both at the same time"(at least not in this view of the world!). In contrast, a computer might not have such an understanding. A human brings their world experience and their understanding of terms such as "man" and "woman" to understanding the representation–something a computer does not do.

The need to capture knowledge with high-fidelity and interpret it unambiguously is enabled by having a representation language with well-defined semantics. In the same way that a C programming language compiler must unambiguously "understand" what each of the language components means in terms of constructing a programme that runs on a particular machine, so must a computer understand what each of the statements in the description of some knowledge represents. This is not the *deeper meaning *of the software (such as typesetting this document according to standard publishing principles) or what, for instance, an ontology is stating about biology. What is unambiguously interpreted is the relationship between the symbols being used. The (computer's) "understanding" is determined by the semantics of the language–be it a programming language or a knowledge representation language. As we will see below, just as a compiler needs to know exactly what a particular programming construct means, though not the intention of the programmer, a computer needs to be able to interpret what the "circles and arrows" mean in Figure 1.

Figure 1 shows, on its right hand side, a simple ontology of **Person**, with two child classes of **Man** and **Woman**. As human users we understand, or believe we understand, what is being represented in such an ontology; "there are two kinds of **Person**, namely **Man** and **Woman**". We can, however, ask several supplementary questions about this ontology:

• Are all instances of **Man** also instances of **Person**?

• Are **Man** and **Woman** the only kinds of **Person** that exist?

• Is it possible for an instance of **Person** to be both a **Man** and a **Woman**?

Now consider the left part of Figure 1 where we say that a **Person** has **Gonads** and that a **Man** has **Testis**. Again, we might ask ourselves several additional questions:

• How many **Testis** does a **Man** have?

• Can a **Man** only have **Testis** or may he have other parts?

• Does having a **Testis** make an instance of **Person** a **Man**?

• Are **Testis** the only gonads a **Man** can have?

• Do all **Man** have **Testis**?

• Are all **Testis** parts of **Man**?

• May I say anything more about the parts that a **Man** has?

Again, as human users of the ontology shown in Figure 1, we may understand, deduce, guess, or know the answers to these questions, or we may not; it is certain, however, that the computer will not do so. It is in the semantics of the knowledge representation language that the answers to such questions can be couched. It is part of the semantics of a language that says whether two children of a concept are overlapping, that is, is it possible for an instance of **Person** to be both a **Man** and a **Woman**. For a computer to know both the answer to this and that the only possible kinds of **Person** are **Man** and **Woman**, this has to either follow from the semantics of "**is-a** arrows" of our formalism, or it would have to be explicitly stated. Remember that the labels are just symbols; the computer does not understand those symbols, but the semantics of the language specifies, for instance, that we have symbols for class names (such as **Man** and **Testis**), that we have symbols for property names (such as **has-part**), and that the **has-part**-labelled arrow from **Man** to **Testis** means that each instance of the class called **Man** is **has-part**-related to at least one instance of the class called **Testis**.

Returning to human users, the semantics of a programming language tells us how a computer will interpret our software, and thus enables us to write software that does what we want it to do. Similarly, the semantics of a knowledge representation language tells us how a computer, a reasoner, or another human should understand the statements in our knowledge base–and a precise semantics tells us this in an unambiguous way.

The semantics might enable a human to interpret a statement as "each and every **Man** has at least one **Testis**", as there is no other interpretation possible; he or she can also bring their world knowledge to decide whether this is true. A user might believe they understand what is represented in the ontology shown in Figure 1, but dangerous assumptions might be made when doing so and this is where ambiguity can occur. If the knowledge representation language has a precise semantics, then the knowledge captured in the ontology expressed in that language can be decoded with precision; that is, we can interpret exactly what each statement in a language means. Precision is vital for humans since it enables them to agree on the meaning of a statement, and for the design of software to take into account a knowledge base since it enables the comparison of what the software actually does with what it is supposed to do *according to the semantics *of the underlying knowledge representation language. For example, a precise semantics allows us to make statements about the ***soundness ***and ***completeness ***of a query answering tool: does it retrieve all and only those answers that *should *be retrieved according to the semantics? This can mean, however, that we need to make an effort to understand the semantics [15].

## 3 OWL

OWL-DL [3] is an ontology language based on ***description logics ***(DLs), which are a family of logic-based knowledge representation formalisms describing "objects", "classes" and the "relationships" between them [16]. Most DLs are fragments of standard ***first order logic***. Originally, they were designed to give a unified logical basis to various well-known traditions of knowledge representation like frame-based systems and semantic networks [17]; they have found various applications in conceptual modelling and as a logical underpinning of ontology languages [16]. OWL-DL is based on an expressive DL, *i. e., *it provides a wealth of constructors to describe complex class expressions from atomic classes and relationships. In this section, we will only use a small portion of OWL-DL's expressiveness to highlight its core features.

The semantics of OWL-DL is best understood when talking about "objects" that are "instances" of "classes", and that are related to other objects *via *"relations".

An object can be an instance of a class, and a class can be a sub-class of another class. For example, the object **Robert** is an instance of the class **Man** which, in turn, is a sub-class of **Person**. The meaning of the sub-class relationship is that all instances of the sub-class, **Man**, are also instances of its super class(es), **Person**. In OWL-DL, to describe a class, we can describe it in terms of other classes (*e.g., *saying that **Man** are "**Person** and not **Woman**") and of properties of its instances.

**Figure 2:**
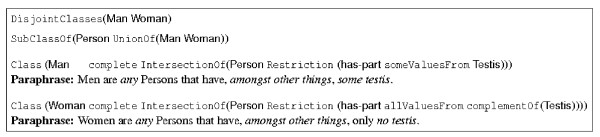
Man and Woman in OWL. Description and paraphrase provided.

In Section 2, we have informally described an ontology with classes **Man**, **Woman**, **Person**, and others. In this section, we will formalize some of these classes in OWL-DL. We start by fixing the relationship between these three classes. First, we declare that **Man** and **Woman** are "disjoint"; that is, it is not possible for an object to be an instance of both classes; this is expressed in the first statement of Figure 2. Similarly, we have to decide whether it is possible for an instance of **Person** to be neither an instance of **Woman** nor of **Man**. Assuming that this is not the case, we add the second statement of Figure 2. Together, these four statements imply that every person is either a man or a woman, but not both.

Next, we make use of OWL-DL's ability to describe a class by describing its superclasses and how its instances are related to other objects. For example, the definition of the class **Man** in Figure 2 states that an instance of **Man** is a (instance of) **Person** which has an instance of **Testis** related to it *via *the **has-part** property. As this statement only says something about the existence of a relationship to another object, it is called an "existential" restriction–which is expressed in OWL-DL using the **someValuesFrom** keyword. This asserts only that an instance of **Man** might have several parts that are testis, and other parts, as well–which is why we use the *"amongst other things" *in the paraphrase. For example, we have left it open in our description of **Man** whether a **Man** has ovaries, and so, with respect to the above definition of **Man**, a **Man** may or may not have ovaries. Additionally, to make this more precise, OWL-DL also allows "universal" restrictions to be made: *e.g., *in the definition of **Woman**, we say that an instance of **Woman** is related *via *the relation **has-part*** only *to instances of the complement of **Testis**, *i.e., *no part of a woman can be an instance of **Testis**. This is expressed using the **allValuesFrom** keyword and **complementOf**, another expressive means which corresponds to logical negation.

**Figure 3:**
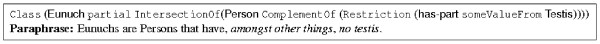
Eunuch in OWL. Description and paraphrase provided.

In the definitions of the classes **Man** and **Woman**, we have used the keyword **complete** to indicate that the following expressions provide necessary and sufficient conditions for an object to be an instance of this class. That is, if we know that **Robert** is a **Man**, we also know that he has a part that is a testis and, if we find a person that has a part which is a testis, then this person is an instance of **Man**. This gives rise to the use of the *"any" *in the paraphrasing used in Figure 2. Replacing **complete** with **partial** means that only the first conclusion can be drawn. For example, Figure 3 contains a partial definition of **Eunuch** as those Persons that do *not *have **Testis**; so every **Eunuch** has no parts which are **Testis**, but not everyone with no **Testis** is a **Eunuch**.

In all of these examples, we have only stated restrictions concerning **Man** and **Woman** and the objects to which they are related by the **has-part** relation. We have not restricted any other relationships we might choose to describe, such as **has-mother**, nor have we said anything about instances of **Testis** apart from the fact that they can be parts of a **Man**. After all, other species' male instances also have **Testis**, *i.e., *according to our ontology so far, an instance of **Testis** can be part of other objects or of nothing at all.

**Figure 4:**
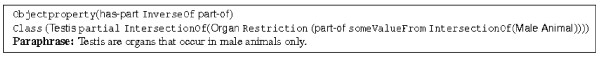
Testis in OWL. Description and paraphrase provided.

In order to avoid such "homeless" testis, we can add a restriction which states that an instance of **Testis** is a part of a male animal. For this to have the desired effect, we also need to state that **has-part** is indeed the inverse of the relation **part-of**. Both statements are found in Figure 4.

Due to its description logic underpinning, OWL-DL ontologies can be submitted to a DL reasoner which provides reasoning services. Most importantly, a reasoner can decide the consistency of each class defined in the ontology and it can compute the implicit class hierarchy. For example, given the statements made so far, the reasoner infers that a **Eunuch** is, in fact, a subclass of **Woman**. This seems a little counter-intuitive, so we might also assert that a **Eunuch** is a subclass of **Man**. The reasoner will then tell us that **Eunuch** is inconsistent: there can be no instances of it. In this case, it is probably our definition of **Man** that is a poor model of reality. The inconsistency of the **Eunuch** forces us to re-examine this model. The precise and explicit nature of models in OWL-DL allows us to check the knowledge we have captured as OWL-DL statements and have them to be interpreted correctly.

For a complete description of OWL-DL, we refer the reader elsewhere [3]. Here, we have only used a small part of OWL-DL's expressiveness. In addition to using a relation in both directions (*e.g., *we have used the inverse direction of **has-part*** via ***part-of**), OWL-DL also enables us to state that a relation such as **part-of** is transitive (*e.g., *making a **SemiNiferousTubule** part of a **Testis** also makes it part of a **Man**) and to restrict the number of objects to which an instance of a class is related by a specific relationship (*e.g., *restricting the number of gonads a **Person** has to 2). It should be enough, however, to indicate that the well-defined semantics of OWL-DL enables both the author and a computer to "understand" precisely what has been stated, and enable software such as a reasoner to deduce implicit knowledge from such representations [18,19].

## 4 GO and DAG

The aim of this section is to elucidate the semantics of GO's encoding and not to examine the correctness of the biology captured in that encoding, which has been done elsewhere [20]. There is need, however, to sometimes look at the biology in order to understand the encoding. In contrast to OWL, the semantics of the representation used by the GO is not based on a logical formalism. Our understanding of the GO DAG's semantics comes from its description in English [21], from consultation with members of the Gene Ontology Consortium, and from inferences made from the ontology itself.

**Figure 5:**
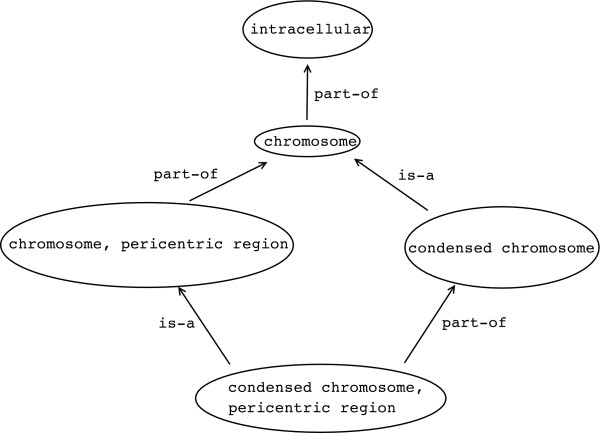
A Gene Ontology Directed Acyclic Graph (DAG). The DAG has both is-a and part-of relationships.

The GO is formalised as a Directed Acyclic Graph (DAG); see Figure 5 for an example of a DAG. More precisely, a "directed graph" is a structure with "nodes" and "edges", the latter being ordered pairs of nodes. In our case, both nodes and edges are "labelled": nodes with the term denoting the class they stand for, and edges with the kind of relationship that relates the corresponding classes. In GO's DAG, edge labels are restricted to **is-a** and **part-of**. Such a graph is acyclic, *i.e., *a DAG, if there is no path *via *edges that relates a node with itself–regardless of the edge's label, but using them only in a "forward" way. The graph in Figure 5 is a DAG, for example. In GO, the term labelling a node refers to this node and all of its children [21].

In addition to this structured knowledge, the GO DAG contains additional information within nodes: a specific GO identifier for each node, as well as "exact", "broad", "narrow" and "related" synonyms for the term labelling a node, and possibly a definition of the meaning of the term. The latter are given in natural language, *i.e., *they are free text descriptions that "define" what a term means. As a consequence, they may come with all the ambiguities of natural language, and we can sometimes not distinguish, for example, between a necessary condition and one that is necessary and sufficient. GO definitions are used by annotators and GO curators alike when using GO, and are not intended to be used by an automated reasoning tool to draw new inferences. The format for the GO DAG also allows for some provenance information, such as author, source, *etc. *but this detail is beyond the scope of this article, where the emphasis is on the main ontological components of the representation.

Next, we discuss what kind of statements can be made in GO's DAG representation. Firstly, GO uses two relationships, **is-a** and **part-of**. Figure 5 shows an example of a GO DAG with both kinds of relationships. The **is-a** relationship points from a child (more specialised) to a parent (more generalised) term [21]. We note that, if a parent has more than one child, there is no way to distinguish between possibly overlapping (*e.g., ***Carnivores** and **Mammal**) and disjoint (*e.g., ***Man** and **Woman**) classes [21]. When interpreting the GO documentation, care should be taken because the **part-of** relationship, in GO's usage, talks about parts and parents, not *parts *and *wholes*, as is ontologically conventional [22]. In Figure 5, we can see what some [13,23] have called "orphan" nodes, *i.e., *a node that is **part-of** another node, but is not a kind of any node. Conventionally, this would be a child with no parent, *i.e., *an orphan, and the GO curators are undertaking an effort to remove such orphans since they indicate an imprecise modeling (personal communication with Amelia Ireland from the Gene Ontology Consortium).

There are (at least) four readings of a **part-of** relationship in GO's DAG [21]. Considering the **part-of** edge from a node labelled **P** to a node labelled **W**, we have the following possibilities:

1. The **part-of** relationship makes no assumption of the existence of the relationship between the nodes in either direction. Any **P** may or may not be part of a **W** and any **W** may or may not have a part that is a **P**. An example is **Person** and **Testis**. Note that this need not contradict the directed nature of the arcs. The **part-of** is directed, but these semantics tell us how it is to be interpreted, particularly with respect to whether such a relationship exists or not.

2. Wherever a **P** exists, it is as part of a **W**, *e.g., ***Nucleus** and **Cell**.

3. Wherever a **W** exists, it has a part that is a **P**, *e.g., ***AvianRedBloodCell** and **Nucleus**.

4. Wherever a **P** exists, it is a part of a **W** and wherever a **W** exists, it has a part that is a **P**. This reading is simply the conjunction of readings 2 and 3. An example of this is **NuclearMembrane** and **Nucleus**.

In the GO documentation [21] the "true path rule" states that "the pathway from a child term all the way up to its top-level parent(s) must always be true". This should be true for both kinds of relationship in GO. For the **is-a** relationship, this means that an individual labelled as **Man** could also legitimately be labelled as **Person** or **Animal**. So, a gene product labelled as a **photoreceptor activity** is also a kind of **signal transducer activity** and finally, a **molecular function**. Thus, the "true path rule", when working along **is-a** relationships implies that we read these relationships in a ***monotonic ***way, *i.e., *every instance of a class is also an instance of its superclasses, without exceptions.

For the **part-of** relationship, this has several implications. Firstly, it means that this relation is assumed to be transitive, *e.g., *if a gene is part of a nucleus which, in turn, is part of a cell, then this gene is part of this cell. This assumption is widely accepted [24]. Secondly, this means that we have to choose one of the readings 2 or 4 mentioned above. The GO editing style guide mentions that the majority of **part-of** links in GO conform to reading 2; readings 1 and 3 are not used as they would violate the true path rule in GO [21].

If we restrict our attention to reading 2, then it is not difficult to verify that the true path rule is even correct when we combine both kinds of relationship in one path: a path using both **is-a** and **part-of** becomes indeed **part-of**. The GO DAG editing style guide warns explicitly against employing a reading different from the second one since such an "abuse" might yield unwanted consequences *via *the true path rule, and suggests that the best strategy is to re-structure GO with new nodes and relationships so that only reading 2 is employed and the true path rule can be employed correctly. As a consequence, while we might have stated that a **Testis** is part of **Man**, we cannot say anything about a **Man** having part **Testis** since this would involve reading 3.

## 5 Reconciling the two representations

In this section we reconcile the semantics of OWL-DL and GO's DAG: we analyse how one can be translated to the other and where, in that process, there could be problems. To perform such a translation it is necessary to understand the semantics of source and target languages and the aim is, of course, to say the same in each representation.

We start by assessing a technical issue that does not affect the semantics, but is important: naming conventions. OWL-DL has got its own naming conventions: non alpha-numeric characters or white spaces are not allowed in the names of the classes, only underscores and alpha-numeric characters. This presents a problem since many GO term names include non-alphanumeric characters. A solution to this problem is to translate any non-alphanumeric character into a string that spells out the disallowed: for example **(-)**-**borneol dehydrogenase activity** in GO would become **PAR_MINUS_PAR_MINUS_borneol_dehydrogenase_activity** in OWL. There is a choice to be made as to whether the term or GO identifier become the class label. The id is the primary identifier (GO:0047503), but the term is the more readable. Whatever the decision, one can be represented using the class label and one using an assertion on an "annotation property": in OWL-DL, we can declare a property to be an annotation property, and then use such a property to attach information to classes–without them being taken into account by an OWL-DL reasoner. That is, assertions on annotation properties act as ***comments ***from a DL point of view, yet they can be displayed to the biologist as a piece of information on this class–just as in GO. The most suitable annotation property for labelling a term with its id is **rdfs:label**, which is already included in OWL.

We cannot translate the natural language definitions associated with a term into OWL-DL axioms. These definitions might be expressible in OWL, yet we cannot automatically generate the correct OWL-DL expressions from a piece of English text. We can, however, capture them using another assertion on an annotation property.

We can capture the synonyms and other alternative labels given for a term in a variety of ways:

1. As assertions on an annotation property;

2. Using equivalence, subclass and superclass axioms;

3. A mixture of approaches one and two.

In the first approach, we can use a series of annotation properties such as exact synonym, broad synonym, narrow synonym and related synonym.

In the second approach, if *S*_1_,...,*S*_*n *_are the exact synonyms given for a term *T*, then we translate this into an equivalence axiom EquivalentClasses(*T S*_1_...*S*_*n*_). Thus, each instance of *S*_*i *_is also an instance of *T *and each *S*_*j *_and, *vice versa*, each instance of *T *is also an instance of each *S*_*i*_.

In OWL-DL, an equivalence axiom EquivalentClasses(*T S*) means that the classes *T*, *S *involved have the same extent of instances. It can further be argued that they are therefore the same class. If the synonyms are exact, this is logically correct, though the ontologist might be presented with a plethora of classes in the user interface. It can be argued, however, that for the user this is simply a presentational issue, and that the user interface should collapse equivalent classes. Some methodologies, such as [25], suggest that a minimal number of classes should be used in an ontology. Use of equivalent classes does not break such an edict if we interpret classes with the same extent of objects as the same class (which is, after all, what is being said). It should be remembered, as is the message all through this article, that the reader should be wary of conflating presentation and the real semantics of a statement. Just as assumptions can be made about the presentation in Figure 1, so can assumptions be made about syntax showing "multiple" classes in an OWL-DL file.

A more significant argument is that this solution conflates a class level argument with a lexical argument. It should be remembered that labels on classes can change, while the class itself is unaltered. One only has to think, for instance, of the different French, German and English words for **Leg** that all refer to the same class of instances. Also, the equivalence axiom approach breaks when the synonyms are not exact synonyms. It could then be argued that the synonym labels should not be used, but one of narrow, broad or related. Hence the equivalence axiom solution is slightly sub-optimal since we would have preferred to have only a single class and more than one name for it, yet this would have required some expressiveness not (yet) available in OWL-DL, and the second approach has largely the same effect. In a similar manner to the equivalence axiom, if we have an alternative name *S *that is "broader than" a term *T*, then we add a statement SubclassOf(*T S*), and if we have an alternative name *S *that is "narrower than" a term *T*, then we add a statement SubclassOf(*S T*).

Please note that the second approach does not take into account related class labels which are not either exact, narrow nor broad, like **virulence** and **pathogenesis**. In this case, we can only suggest to use the first approach. In approach two, we cannot completely translate all class labels in an OWL-DL form, because the **related-to** tag has no reasonable representation as either subclass axiom or restriction upon a class, so we would have to use approach three, with a mixture of logical axioms and one assertion on an annotation property.

The use of the extra equivalence and subclass axioms has a logical argument and can be useful. When a reasoner is applied to such a translation, inconsistencies can be found. If the translator, however, feels that this approach mixes lexical and logical issues then only approach 1, using only assertions on annotation properties is the most valid approach.

Next, the DAG **is-a** relationship translates directly into OWL's sub-class relationship since they have the same semantics, *i.e., *every instance of a class is also an instance of each of its superclasses.

We can assume that subclasses in the DAG representation, like OWL subclasses, overlap by default, *i.e., *if *C*_1 _and *C*_2 _are subclasses of the same superclass, then we cannot exclude that there exists an object that is an instance of both *C*_1 _and *C*_2_. This will capture most of the biology in GO correctly. However, we might want to examine the GO and check, for each pair of subclasses, whether we cannot provide more information. For example, we should ask ourselves whether it is possible for an individual molecular function to be both function-x and function-y at the same time. If this is not the case, then we should make this knowledge explicit in the OWL ontology through the axiom DisjointClasses(**function-x function-y**).

In a similar manual step, we should add covering constraints where appropriate. A covering axiom means that, if an object is a member of a class, then it must be a member of one of the classes that it is asserted to "cover". That is, if **Person** covers **Man** and **Woman**, then any object that is a **Person** must be either a member of **Man** or a member of **Woman**, but it is possible not to have enough information to know to which of these classes that object belongs. For a biological example, if **Enzyme activity** covers all the enzyme functions, then an enzyme activity must be one of those activities; a new enzyme activity would be inconsistent with the ontology. The GO DAG representation does not allow such axioms and we believe that biologists would not use them widely even if it were possible because such axioms would require more knowledge than is usually available. An assumption of no covering is, therefore, not unreasonable.

Since the GO DAG does not capture disjointness or covering constraints, its inclusion is a matter of capturing biological knowledge, and there is no way of simply automating knowledge of disjointness. An automatic translation is possible if it is assumed that there is no "covering" and all sibling classes can possibly overlap.

### 5.1 Capturing the GO DAG part-of in OWL

OWL-DL provides a language that allows us to use as many properties as we want, and we can constrain their interpretation in a number of ways using existential, universal, or cardinality restrictions, and we can make statements about them such as one property being implied by another one or that a property is transitive. In Section 4, we have discussed four possible readings of the GO DAG's **part-of** links, and we show here how these different interpretations can be captured *via *translations to OWL-DL axioms. The advantage here is that, rather than using a single construct which may be read in a number of different ways, OWL-DL allows us to distinguish between these different readings. We can then use different readings of the **part-of** relationship (*e.g., *those discussed in Section 4), without any danger of confusion. In the following examples, we consider how we capture the particular semantics of the assertion **P part-of W**.

**Reading 1 **does not impose any restrictions on an instance of **P** or **W** as they only deal with the *potential *for the relationship. If one insists, one can translate this reading into an OWL-DL axiom

SubClassOf (P UnionOf ((restriction(part-of someValuesFrom W))

ComplementOf (restriction(part-of someValuesFrom W))),

yet this statement does not impose any constraints: indeed, it is equivalent to saying that **P** is a subclass of OWL:thing or saying nothing. In contrast, impossibilities do impose constraints, and we can express them in OWL-DL: to express that a **P** can never be part of a **W**, we can add the OWL-DL axiom

SubClassOf (P ComplementOf (restriction(part-ofallValuesFromW)))

Reading 2 Whenever a P exists, it is part of a W. This can be represented through the following axiom:

SubClassOf(P restriction(part-of someValuesFrom W)),

stating that, for each and every instance of **P**, there *must *be an instance of **W** of which it is a part. For example, every instance of **SemiNiferousTubule** is a part of an instance of **Testis**.

**Reading 3 **Whenever a **W** exists, it has some **P** as a part. This can be represented through the following axiom:

SubClassOf (W restriction(has-part someValuesFrom P)),

provided that we have declared that the property **has-part** is the inverse of **part-of**, as in Figure 4 (many description logics allow the definition and use of inverse relationships; in OWL there is no inverse property *operator *for use in expressions, but we can introduce and define properties as inverses). Inverse properties are interpreted as one would expect: two individuals *a *and *b *are related *via *a property *P *if and only if *b *and *a *are related *via *the inverse of *P*. For example, we can use such an axiom to state that every instance of **Testis** has a part that is an instance of **SemiNiferousTubule**. Please note that this statement and the one given as an example for the third reading are independent in the sense that they do not imply each other.

**Reading 4** This is simply a conjunction of 2 and 3, and we can thus encode it by including both axioms introduced above.

As mentioned before, GO employs reading 2 for **part-of** links. Hence we translate each such link into the corresponding OWL statement. Additionally, we can then manually add more statements, *e.g., *in cases where our biology tells us that reading 4 would be more precise. These various semantics for the **part-of** relationship used in the GO DAG pre-date the OBO relationships described below in Section 6. In the OBO relationships, as we shall see, the semantics are more strictly defined and the translation to an existential property on a class, as in interpretation above, is clear.

Recall that, in GO, orphan nodes are those that do not have any outgoing **is-a** link. In OWL-DL, the corresponding classes do not cause any problems since they will be automatically placed in the class hierarchy under the most general class called **OWL:thing**. There are, therefore, no orphan nodes in an OWL-DL ontology and any modelling that makes any biological assertions to overcome placing subclass axioms to **OWL:thing** must be part of a process independent of the translation of representation.

That completes our discussion of the translation of GO's DAG into OWL-DL. We can see, therefore, that it is possible to represent what is captured in the GO in OWL-DL with making only two assumptions, both of which are reasonable. The OWL-DL representation will capture the same knowledge as the GO DAG. In addition, we can even distinguish between the uses of readings two and four in the **part-of** relationship in GO.

### 5.2 Translating OWL-DL back into DAG

As we have observed above, the DAG's **is-a** relationship and the subclass relationship in an OWL-DL ontology have the same reading. Hence we can take an OWL-DL representation of a DAG ontology, ask a reasoner such as FaCT++, Pellet or Racer [26-28] to infer all subclass relationships, and then translate the resulting class hierarchy back into DAG format. To be more precise, for each class name *A*, we first ask the reasoner to return all classes that are equivalent (if we have used the translation of synonyms using equivalence axioms described above) with *A*. Then we choose a "main" node label *A' *from *A *and the reasoner's answer, and create a node labelled *A' *whose exact synonyms are set to *A *(in case that *A' *is different from *A*) and the reasoner's answer (possibly minus *A*'). As a result of this step, we obtain a set of nodes labelled with terms and exact synonyms. Next, for each pair of node labels *A*, *B*, we ask the reasoner whether *A *is a subclass of *B*. If this is the case, we add an **is-a** link from *A *to *B*, otherwise we do not do anything. Similarly, for each pair of node labels *A*, *B*, we ask the reasoner whether *A *is a subclass of restriction(**part-of** someValuesFrom B). If this is the case, we add a **part-of** link from *A *to *B*, otherwise we do not do anything. Narrow and broad synonyms can be obtained by looking for subclasses and superclasses, respectively, yet this would be exactly the same information as represented in the **is-a** structure and thus redundant. Finally, those features of the GO DAG that we have translated to assertions on annotation properties can be retrieved and back-translated appropriately.

As a result, we obtain a graph whose nodes are labelled with names and sets of synonyms, and whose edges are labelled with **is-a** and **part-of**. If any axioms have been added to the GO in OWL, such as disjointness or covering axioms, these are retrieved through calls to the reasoner. Disjointness can be represented in the OBO format (see Section 6 below), but covering cannot. So, the back-translation of an augmented GO in OWL might well be lossy; *i.e., *they are lost in translation. This would also be true of all those features of OWL-DL that cannot be expressed in the OBO format. In general, this graph might not necessarily be acyclic, *i.e., *it may contain cycles. Since the GO DAG only allows **part-of** and not **has-part** relationships, however, common sense tells us that we should obtain an acyclic graph: a cycle would need to involve a **part-of** link since pure **is-a** cycles have been collapsed into a single node by construction. Now a cycle involving a **part-of** link, say from a node labelled A, would mean that, in every world conforming to our ontology, we have an infinite chain of instances *a*_*i *_of *A *with *a*_1_** part-of ***a*_2 _**part-of ***a*_3 _**part-of**..., which clearly clashes with our intuition. However, if other relationships are used in the DAG, such as **has-location** or **interacts-with**, a cycle could easily arise (*e.g. *a protein that interacts with itself). As we will see below (Section 6) the wider OBO language allows cycles.

## 6 Representing other OBO relationships in OWL

Open biomedical Ontologies (OBO [5]) is a collection of bio-ontologies, and they come with a core set of biological properties for use within OBO ontologies [29,30]. The aim is to have consistent interpretation and use of properties representing biological relationships. Here we describe what aspects of the OBO relationships can be represented in OWL. The OBO relationships talk about properties at three levels, and we can easily distinguish these relationships in OWL:

1. Stating that an individual a is an instance of a class *C*: this is expressed by

Individual(*a *type* C*)

2. Stating that an individual *a *is related *via *a certain property *P *with an individual *b*: this is expressed by

Individual(*a ***P ***b*)

3. Stating that each instance of a class *C *is related *via *a property *P *to some instance of a class *D*.

SubClassOf(C restriction(**P **someValuesFrom** D**))

In our experience, the third of the above relationships is the most commonly used in building ontologies based on classes, where the (all – some) form of definition used in the OBO relationships fits perfectly with the DL style of relationships [31]. The OBO relationships, so far at least, are therefore readily mapped to a restriction with existential quantification. The OBO relationships are of the third kind, but built upon a series of primitive relationships between instances such as **part-of** holding between two continuants; **located-in** holding between a continuant and a region; **derives-from** holding between two continuants; *etc*.

As mentioned, these basic OBO relationships can be expressed in OWL. In OBO, they are extended, however, to take into account both temporal and spatial aspects, none of which can be expressed in OWL: for example, OWL allows us to state that every instance of *C *must be **located-in** an instance of *D*, yet we can not express that an instance of *C*' must *eventually *be **located-in** an instance of *D *or that an instance of *E *will *eventually *be an instance of a class *F*. For example, we might want to express that **Adult** is –a–cont** child** because an instance of the class **Adult** has *at some previous time point *been a **Child**. Since OWL-DL takes an entirely static view of the world, such a statement cannot be made in OWL. There are extensions of (the DL underlying) OWL-DL that can deal with these temporal aspects [32], but the reasoners used for OWL-DL do not handle these logics nor does the OWL-DL syntax or semantics accommodate these temporal aspects.

The OBO file format includes several aspects that should be translated into OWL; some of them are required aspects, others are optional, see Table 1. Some of these aspects have already been analysed in this paper for the GO's DAG: the synonyms (**related_synonym**, **exact_synonym**, **broad_synonym** and **narrow_synonym**), is_a (sub-class relationship in OWL), **relationship** (existential restrictions in OWL, as already described in the case of **part-of**) and **is_transitive** (transitive properties in OWL). For the rest, the translation is provided in Table 1. It is intended that the OBO language has the same semantics as OWL (personal communication with Chris Mungall from the Gene Ontology Consortium) and this is the approach we have adopted, though the documentation was at times unclear.

**Table 1:** Translation of OBO aspects into OWL.

**OBO stanza**	**OWL**	Required/Optional
name	OWL-DL class name	required
id	Extra-logical	required
alt_id	Extra-logical	optional
namespace	OWL namespace	optional
definition	Extra-logical	optional
comment	Extra-logical	optional
subset	Extra-logical	optional
related_synonym	"Some values from" restriction on related_synonym	optional
exact_synonym	Equivalent class	optional
broad_synonym	Superclass	optional
narrow_synonym	Subclass	optional
xref_analog	Extra-logical	optional
xref_unknown	Extra-logical	optional
is_a	Subclass	optional
relationship	"Some values from" restriction on object property	optional
is_obsolete	Extra-logical	optional
use_term	Object property	optional
domain	domain	optional
range	range	optional
is_cyclic	(see Section 6)	optional
is_transitive	transitive	optional
is_symmetric	symmetric	optional

In Table 1, many of the OBO entries are described as being "extra-logical" in OWL-DL. This means that they are not part of the descriptions of the objects in a class. For instance, a GO id is a description of the class, not a description of the instances or objects of that class. OWL-DL can currently only represent these extra-logical aspects with the annotation properties mentioned earlier in Section 5.

The OBO optional tag **is_cyclic** is intended to convey that a relationship can be used to form cycles (such as, **interacts-with** forming cycles of interacting proteins). Properties in OWL-DL are inherently free to do this and so **is_cyclic** could only be preserved as another annotation property.

The OBO file format allows for property hierarchies (for example in the Sequence Ontology [33]), but the DAG does not use them. For the wider OBO representation, it is perfectly possible to translate property hierarchies.

## 7 Implementation

This translation has been implemented as a Java (1.5) programme [34]. It takes a GO ontology expressed in the OBO 1.0 format [8] and produces the same ontology expressed in OWL DL in the RDF/XML syntax [35].

The OBO flat file is read and the WonderWeb OWL API [36] is used to create the OWL-DL ontology. The OBO flat file is parsed and loaded into memory as an intermediate representation that is later explored by the programme and each OBO element is used, as appropriate, to supply parameters to operations on the WonderWeb API. For example, two class identifiers are supplied to the subclass operation to create an OWL subclass axiom; the id of the OBO term is supplied as a value for **rdfs:label** property; the second half of the OBO **part-of** relationship is supplied as a filler for an existential restriction on the **part-of** object property; and so forth. Finally a new OWL file in RDF/XML syntax is created on the hard disk.

The translation uses the approach 2 to deal with synonyms: exact synonyms, narrow synonyms and broad synonyms are translated as new equivalent classes, subclasses and superclasses, respectively.

## 8 Discussion

In this paper we have attempted to convey why computer scientists seem to care so much about a representation language's semantics. In essence, it is in order to prevent ambiguity of interpretation (at a far simpler level than intention) of statements in the language. We only have to think back to our toy informal ontology for **Man** and **Woman** to see the trouble that imprecision can give. One only has to substitute genes, proteins, processes, etc. into these types of informal statement to realise the wide variety of interpretations that can be placed on ill-defined statements. "P53 activates transcription"–is all transcription activated by P53? do all P53 activate transcription? In this sense formality is very useful. OWL is, of course, not the only formal language. Our point here is, however, to exemplify the benefits of such formality in communicating to humans and computers.

By examining the semantics of OWL-DL and GO's DAG, we have seen that converting from GO DAG to OWL-DL presents no real problem, as long as we are willing to make assumptions on disjointness and covering. We can even translate our OWL-DL ontology back into DAG. There are various benefits of having an OWL-DL version of a DAG ontology. Firstly, we can say things in OWL-DL that we cannot say in DAG, and we can thus make properties and relations explicit. For example, we can express covering between classes, we can use properties in both directions, and we can formulate necessary and sufficient definitions of classes. Secondly, these statements are amenable to machine interpretation: that is, we can have an OWL-DL reasoner classify our ontology and detect inconsistent classes. This can help us find modeling errors in our ontology, *e.g., *when the reasoner comes back with un-intended inferred **is-a** links or inconsistent classes, and thus supports the design of a *good *ontology. Thirdly, we can annotate documents with complex OWL-DL class descriptions and have the reasoner take these into account when answering queries. That is, we are no longer restricted to the classes present in the ontology, but we can make them up on-the-fly *and *have these descriptions taken into account for query answering. In DL style ontologies, it is common for classes defined in an ontology to be the building blocks of other classes, rather than enumerating all the possible classes. Obviously, converting an ontology that comes with such expressiveness and inference services to one that lacks them might lead to an impoverished ontology. Fourthly, we can extend our translations in a straightforward way to other OBO relations.

In summary, we have described the role of a language's semantics. We have also described the role and benefits of a representation language with well-defined semantics and reasoning support. The core of the argument is that, if ontologies are to fulfill their role of providing a common, shared understanding of a knowledge domain, then the statements within that ontology have to be able to be interpreted unambiguously. We then examined the semantics of GO's DAG and compared it with OWL-DL. Our results of examining the expressive means provided by both formalisms and their semantics is that conversions between the OBO representation and the GO DAG subset is possible (within some constraints) and leads to interesting new possibilities.

## 9 Authors' contributions

All authors contributed both writing and thought to this paper. MEA led this effort. RS, SB, PL and US produced an early draft and MEA brought it to conclusion, including the implementation. US and SB provided expertise on OWL and description logics. All authors read and approved the final manuscript.

## Supplementary Material

Additional File 1 "Glossary". list of computer science terms used in this article, with their definitions. Click here for file
