# Supplementary material for: Understanding and using the meaning of statements in a bio-ontology: recasting the Gene Ontology in OWL
Source: BMC Bioinformatics. 2007 Feb 20;8:57. doi: 10.1186/1471-2105-8-57 (PMC1819394; doi:10.1186/1471-2105-8-57)
Supplement: Additional File 1 — "Glossary". list of computer science terms used in this article, with their definitions. [file 1471-2105-8-57-S1.pdf]

GLOSSARY ("Understanding and using the meaning of statements in a bio-ontology: recasting the Gene Ontology in OWL")

Mikel Egaña Aranguren[1], Sean Bechhofer[1], Phillip Lord[2], Ulrike Sattler[1], Robert Stevens*[1]

[1]School of Computer Science, University of Manchester, Manchester, United Kingdom
[2]School of Computing Science, University of Newcastle, Newcastle, United Kingdom

Email: Robert Stevens - *– robert.stevens@manchester.ac.uk, Mikel Egaña Aranguren - mikel.eganaaranguren@stud.man.ac.uk, Sean Bechhofer - sean.bechhofer@manchester.ac.uk, Phillip Lord - phillip.lord@newcastle.ac.uk, Ulrike Sattler - sattler@cs.man.ac.uk;

*Corresponding author

Knowledge Representation Language: A language used to represent information about the world or a particular domain. Knowledge Representation Languages are often formal languages (with a precise meaning or semantics for the operators in the language), allowing for the use of deduction or reasoning (see Reasoning).

Reasoning: The application of a set of rules or processes in order to derive conclusions from a set of statements. For example, "consistency checking" can determine whether a set of facts are self-consistent or contains a contradiction. Reasoning can be used to realize a number of tasks in Knowledge Representation, for example computing implicit classification hierarchies from collections of definitions or query answering.

Knowledge Base: An ontology populated with instance data in addition to class definitions.

Soundness: Soundness is a property of an inference procedure or proof theory for a language that is in terms of the underlying semantics of the language. A Sound procedure is one which is guaranteed to only produce correct conclusions. See also Completeness.

Completeness: Completeness relates an inference procedure or proof theory for a language with the underlying semantics of the language. A Complete procedure is one which is guaranteed to find all valid inferences. See also Soundness.

Description Logics: Description logics are a family of Knowledge Representation Languages tailored for expressing knowledge about concepts and concept hierarchies. They are usually given a declarative semantics, which allows them to be seen as sub-languages of predicate logic. They are considered an important formalism unifying and giving a logical basis to the well known traditions of frame-based systems, semantic networks and KL-ONE-like languages, object-oriented representations, semantic data models, and type systems. Description Logics systems have been used for building a variety of applications including conceptual modeling, information integration, query mechanisms, view maintenance, software management systems, planning systems, configuration systems, and natural language understanding. In general, DL languages are "well-behaved", with sound and complete procedures for inference. See also Soundness, Completeness.

First Order Logic: First Order Logic (FOL or First Order Predicate Calculus or Predicate Logic) is a formalism that allows us to describe the properties of objects and relationships between them. FOL has a well-defined syntax (a way of writing statements down), a semantics (an unambiguous specification that tells us how to interpret those statements) and proof theory (a set of rules for deriving conclusions from a set of hypotheses).

Monotonic: In a monotonic logic, conclusions can not be falsified by the addition of new information. In a non-monotonic logic, in contrast, the addition of new hypotheses can cause previously derived conclusions to become false.

Comments: Comments in software source code are annotations that are primarily intended for humans. They are not usually interpreted by the computer, *i.e.* they have no impact on the execution of the code.
